# The Safety and Effectiveness of Root ZX3 for Symptomatic Irreversible Pulpitis: A Protocol for a Single-Center, Open-Label, Single-Arm Clinical Trial

**DOI:** 10.7759/cureus.104171

**Published:** 2026-02-24

**Authors:** Takashi Matsuura, Asuka Aka, Hidetaka Ishizaki, Atsutoshi Yoshimura

**Affiliations:** 1 Department of Periodontology and Endodontology, Nagasaki University Graduate School of Biomedical Sciences, Nagasaki, JPN

**Keywords:** clinical trial, high-frequency current, pulpectomy, root zx3, study protocol, symptomatic irreversible pulpitis

## Abstract

Background

Root ZX3, a high-frequency dental generator introduced in 2021, utilizes high-frequency current (HFC) within the root canal to coagulate and incinerate pulp tissue in seconds. Despite its clinical potential, evidence regarding its specific application for symptomatic irreversible pulpitis (SIP) remains scarce. This study aims to evaluate the safety and clinical effectiveness of Root ZX3 for the management of SIP in a dedicated clinical trial. Specifically, the primary objective is to assess the immediate relief of spontaneous pain (primary endpoint) and the incidence of adverse events following HFC application.

Methods

In this single-center, single-arm trial, we will include 10 permanent teeth diagnosed with SIP. Following infiltration anesthesia and dental dam isolation, an access cavity will be prepared to allow the Root ZX3 tip electrode to contact the pulp tissue. After the HFC application, the cavity will be sealed with a temporary restoration. Our primary endpoint is the resolution of spontaneous pain on postoperative day 0, defined as the proportion of participants achieving complete relief.

Discussion

SIP-related emergencies often disrupt clinical workflows due to their unscheduled nature. The treatment time is a critical bottleneck in these scenarios. By employing Root ZX3, clinicians may manage SIP more efficiently without increasing chair time or affecting other patients. If this protocol proves effective and safe, Root ZX3 will offer a valuable technical alternative for the immediate management of acute pulpitis.

Conclusion

This clinical trial will provide the first systematic evaluation of the Root ZX3’s HFC application for managing SIP. By leveraging its ability to rapidly coagulate pulp tissue in anatomically complex areas, this approach aims to streamline the emergency endodontic workflow without compromising patient safety or treatment precision. Although this exploratory study is limited by its single-center design and small cohort, the results are expected to provide a critical foundation for optimizing pain-relief protocols. Ultimately, demonstrating the effectiveness of Root ZX3 could establish it as a valuable, time-efficient option for the immediate relief of acute pulpal pain in busy clinical settings.

## Introduction

Pulpectomy is one of the most common treatments for symptomatic irreversible pulpitis (SIP). According to clinical guidelines published in the Journal of Endodontics, pulpectomy remains the primary treatment for SIP, alleviating symptoms such as spontaneous pain, percussion pain, and others [1]. This approach is supported by the American Association of Endodontists' position statements on treatment planning for endodontic cases [2,3]. In clinical practice, many patients with SIP present without an appointment, requiring their emergency treatment to be performed within an already busy clinical schedule. It can be challenging to completely remove dental pulp tissue due to the complex anatomy of the root canal system. Residual pulp tissue after pulpectomy may cause postoperative pain, including occlusal pain, percussion pain, or pain during root canal preparation. Furthermore, performing root canal treatment within limited time frames may increase the risk of complications, such as pulp floor perforation, ledge formation, or file fracture, due to the need to rush the procedure. Deviations in root canal morphology, such as perforations or ledges, may complicate subsequent root canal treatment and could adversely affect the outcomes of retreatment should it become necessary [4,5]. Additionally, although preservation of peri-cervical dentine and minimally invasive root canal treatment are increasingly emphasized, root canal treatment undertaken under time pressure may also lead to excessive removal of cervical dentine [6].

In 2021, J. Morita Manufacturing (Kyoto, Japan) launched the Root ZX3, a next-generation apex locator [7]. While it serves primarily as a high-precision device for root canal measurement, it features an optional high-frequency module capable of generating high-frequency current (HFC; 530 kHz). By attaching this module, the device can utilize HFC within the root canal to coagulate and incinerate dental pulp tissue within a few seconds. The device can coagulate and incinerate pulp tissue even in complex, narrow root canals that cannot be entirely removed by mechanical instrumentation; however, clinical evidence regarding the use of Root ZX3 for the management of SIP is lacking. To assess the safety and efficacy of the Root ZX3 in managing SIP, this single-center, single-arm clinical study was developed.

## Materials and methods

Trial design

This is a single-center, open-label, single-arm clinical trial. The PICO (Population, Intervention, Comparison, and Outcome) framework for this study is summarized in Table 1. To ensure comprehensive reporting, this protocol adheres to the Standard Protocol Items: Recommendations for Interventional Trials (SPIRIT) criteria [8]. We have included the finalized SPIRIT checklist in the Appendix.

**Table 1:** PICO question SIP: symptomatic irreversible pulpitis; HFC: high-frequency current

Criteria	Description
P (Participants)	Patients with a tooth diagnosed with SIP
I (Intervention)	Application of HFC
C (Control)	Not applicable (single-arm trial)
O (Outcome)	Proportion of spontaneous pain resolved after intervention

Participant timeline

The schedule of enrollment, interventions, and assessments is summarized in Table 2.

**Table 2:** SPIRIT figure

Timepoint	Enrolment &	Assessment 1	Assessment 2
	intervention	(Day 1, 2, 3, or 7	(Two weeks after intervention
	(Day 0)	after intervention)	range ±7 days)
Enrolment			
Eligibility screening	X	―	―
Informed consent	X	―	―
Intervention			
Intervention	X	―	―
Clinical assessment			
Inspection	X	―	X
Pain during treatment	―	―	X
Percussion	X	―	X
Adverse events	X	―	X
Questionnaire survey	―	X	―

Study setting

All clinical trial activities will take place at Nagasaki University Hospital in Nagasaki, Japan.

Sample size

As the clinical application of Root ZX3 for SIP lacks systematic evaluation, no preliminary data are available for a formal power calculation. This study is therefore designed as an exploratory, single-arm feasibility trial to obtain initial estimates of safety, effect size, and clinical variability.

A total sample size of 10 participants was selected based on feasibility requirements for early-phase device investigations, in which small cohorts (typically 10-20) were appropriate for identifying procedural concerns before larger trials. Given the absence of prior effect-size estimates for immediate postoperative pain relief with this device, a formal calculation would lack a meaningful basis. Data from these 10 cases will provide the necessary preliminary estimates to determine the power and sample size for future multicenter randomized controlled trials.

Eligibility screening

Eligibility will be confirmed by operators in accordance with the criteria listed in Table 3.

**Table 3:** Eligibility criteria SIP: symptomatic irreversible pulpitis; VAS: visual analog scale

Inclusion criteria	Exclusion criteria
Participants with a tooth	Participants with pacemakers or implantable cardioverter-defibrillators
diagnosed with SIP requiring pulpectomy	
Permanent teeth	
Teeth with spontaneous pain	
Teeth with a positive response to an electric pulp test	
Participants aged ≥18 years	
Participants who can complete the VAS assessment independently	
Teeth without periapical lesions	
Periodontal pocket depth of the target tooth <4 mm	
Absence of furcation lesions in the target tooth	
Alveolar bone resorption of the target tooth	
< one-third of the root length	
Participants who provide written informed consent	

Intervention

After enrollment, the following procedure will be performed by operators. Following infiltration anesthesia, the targeted tooth will be isolated with a dental dam, and an access cavity will be prepared in the SIP tooth's pulp chamber using a rotary cutting instrument. Only minimal pulp exposure will be created to allow insertion of the electrode tip. The tip electrode of Root ZX3 was then inserted into the pulp tissue, and HFC (Mode 4; 530 kHz) was applied three times. The access cavity will be sealed with a temporary restorative material (Caviton; GC Dental Industrial Corporation, Tokyo, Japan). Finally, a questionnaire assessing post-treatment pain will be provided to the patient.

If the target tooth presents with extensive caries and severe structural loss, it will be temporarily built up with composite resin to facilitate isolation and treatment; thereafter, the above intervention will be carried out. Any other usage of high-frequency electrosurgery instruments during treatment with Root ZX3 will be prohibited.

At 2 ± 1 weeks after the intervention, the pain questionnaires will be collected, and horizontal and vertical percussion was evaluated by operators. Then, pulpectomy was performed without infiltration anesthesia to assess pain during treatment. After the study, procedures are completed, participants were followed up as part of routine care, and appropriate treatment was provided as needed.

All intervention procedures will be documented in the electronic medical record by the operators to enhance protocol adherence. Criteria for halting the intervention include a participant’s request to withdraw or modify their assigned treatment, any clinical deterioration or onset of comorbid conditions, and any situation where the research team deems continued participation unsuitable.

Primary endpoint

The primary endpoint is the relief of spontaneous pain on postoperative day 0. The primary outcome will be expressed as the proportion of participants in whom spontaneous pain has completely resolved on postoperative day 0.

Secondary endpoints

The secondary endpoints are: (1) Relief of spontaneous pain on postoperative days 1, 2, 3, and 7; (2) Improvement in occlusal pain on postoperative days 0, 1, 2, 3, and 7; (3) Pain during pulpectomy was experienced two weeks after the intervention; (4) Improvement in percussion pain two weeks after intervention.

Safety endpoints

For devices in this category, the following adverse events require particular attention: burns, convulsions, muscle contractions, tissue and mucosal injury and inflammation, hematoma, electric shock, cardiac arrest, and perforation. To date, such adverse events have not been reported with Root ZX3. All adverse events will be recorded and assessed for severity and causality.

Data collection

After the intervention, participants will be given a pain diary to record their pain levels and the dose and frequency of any analgesic medication taken. Spontaneous, occlusal, and percussion pain will be evaluated using a questionnaire. Postoperative Pain Assessment on postoperative day 0 was conducted after the effects of local anesthesia had completely worn off, typically 4 to 6 hours following the procedure. Occlusal and percussion pain will be assessed via a 100 mm visual analog scale (VAS; endpoints defined as 100 for unbearable pain and 0 for no pain), and participants will be instructed to indicate their pain level by marking the VAS line. Operators will assess pain during pulpectomy, which will be performed two weeks after the intervention. For percussion testing, clinical operators will conduct both vertical and horizontal percussion using the blunt end of a dental mirror handle to evaluate the status of the periodontal ligament and periapical tissues. To ensure force consistency and provide a baseline for the participant, the test is first applied to an adjacent or contralateral healthy tooth with a gentle, controlled tapping force. The participant’s response is then recorded using a VAS. These assessments are conducted at baseline (Day 0) and at the two-week follow-up visit.

Data management

Upon obtaining written informed consent, the operator will complete the identification and case registration forms. These documents are subsequently integrated into the electronic medical record and forwarded to the Principal Investigator (PI), who maintains them within the secure research file. To ensure participant confidentiality, a unique research identification number, consisting of a numeric code and a symbol, is assigned to each participant upon enrollment. This identifier is deliberately kept separate from any personal information, such as initials or medical record IDs, that could directly identify an individual.

All study-related documentation, including case report forms, will be managed exclusively using these identification numbers to maintain anonymity. While the PI utilizes these codes for general data handling, a master identification list, linking names and medical record IDs to their respective study codes, will be maintained separately. This list is stored in a secure, lockable cabinet for a minimum of five years post-study, ensuring that participant identification remains possible only when strictly necessary and authorized.

Statistical design

The proportion of participants in whom spontaneous pain is resolved on postoperative days 0, 1, 2, 3, and 7 will be calculated using all enrolled cases as the denominator and the number of participants whose spontaneous pain has resolved as the numerator. The mean, standard deviation, median, minimum, maximum, and quartiles of preoperative and postoperative occlusal pain (VAS values) were calculated on days 0, 1, 2, 3, and 7, and those of percussion-induced pain (VAS values) will be calculated at 2 ± 1 weeks postoperatively. Fisher's exact test will analyze the presence or absence of occlusal pain (based on VAS values) on postoperative days 0, 1, 2, 3, and 7, as well as the presence or absence of improvement in percussion-induced pain (based on VAS values) at 2 ± 1 weeks postoperatively, compared with baseline. The proportion of participants with pain at 2 ± 1 weeks postoperatively will be calculated by using all cases as the denominator and the number of participants with pain as the numerator.

Participants who discontinue participation in the study will be excluded from the efficacy analysis. Data with missing values are not discarded and are subject to statistical analysis.

Access to data

The final dataset generated by this study will be accessible exclusively to the PI. Direct access for other research personnel is not permitted under this protocol.

Monitoring

A designated research staff member will conduct monitoring based on standard operating procedures, delivering findings to the PI within 14 days of the evaluation. Should any adverse events or unforeseen complications arise, the PI will provide medical intervention, notify the CRB, and brief the study team. Given that Root ZX3 has already received approval from the Pharmaceuticals and Medical Devices Agency, a separate data monitoring committee is not required.

Potential benefits and harms

Reducing treatment time may lessen the burden on both participants and operators. If this study demonstrates that Root ZX3 alone is effective in managing SIP, it may expand treatment options and potentially benefit a wide range of patients. Possible side effects of the intervention include: (i) burns; (ii) spasms and muscle contractions; (iii) tissue and mucous membrane damage and inflammation; (iv) tissue injury; (v) burns or injury to other parts of the body of the participants due to improper contact with the counter-rod; (vi) hematoma; (vii) hypersensitivity to electric stimulation; (viii) cardiac arrest; (ix) iatrogenic injury to surrounding tissues; and (x) perforation.

Potential device malfunctions include: (i) ignition or explosion of flammable substances or gases; (ii) unintended output; (iii) unintended power increase or configuration change; (iv) malfunction due to electromagnetic interference; (v) malfunction due to wire breakage; (vi) malfunction due to poor contact; (vii) high-frequency shunt or leakage; (viii) interference with other equipment due to electromagnetic wave; and (ix) malfunction of an implantable cardiac pacemaker or implantable defibrillator. All such risks will be explained to participants during the consent process.

## Results

Trial status

This study (protocol version 1.2.0, approved on 5 March 2025) is ongoing. Participant recruitment began in August 2025 and is planned to continue until December 2027. No interim analysis is planned for this study. Once the study has concluded, the dissemination of our results will align with the Consolidated Standards of Reporting Trials (CONSORT) framework.

Anticipated results

We hypothesize that the application of HFC via Root ZX3 will lead to a high proportion of complete relief from spontaneous pain on postoperative day 0. Since HFC can rapidly coagulate and incinerate pulp tissue even in anatomically complex or narrow areas that are difficult to reach with conventional mechanical instrumentation, we expect a significant reduction in the risk of residual pulp-induced postoperative pain. Furthermore, as this technique is designed to be completed in a few seconds, we anticipate that it will demonstrate clinical feasibility as a time-efficient alternative for managing SIP in emergency settings without the need for prolonged chair time.

Dissemination plan

To maintain a high standard of transparency and reporting quality, the results of this investigation will be documented following the CONSORT criteria. We plan to disseminate the results through presentations at relevant national and international endodontic or dental research conferences. Ultimately, upon completion, the findings from this investigation will be prepared for dissemination in a reputable, peer-reviewed medical journal to provide a foundation for future multicenter randomized controlled trials. Any significant modifications to the protocol will be updated on the Japan Registry of Clinical Trials (jRCT) website to maintain public access to the study's progress.

## Discussion

When a patient presents with spontaneous pain or sharp, persistent pain induced by thermal stimuli, the diagnosis is typically SIP, and pulpectomy is often performed [9]. However, histological findings by Bergenholtz et al. indicate that even in cases diagnosed as irreversible pulpitis, bacterial infection and accumulation of inflammatory cells may be confined to a portion of the coronal pulp. At the same time, other areas contain healthy tissue [10]. Wolters et al. proposed a new diagnostic system and terminology to emphasize the healing potential of the dental pulp, as pulps with reversible inflammation may currently be classified as irreversibly inflamed [11]. Thus, some cases previously diagnosed as SIP may, in fact, be suitable for vital pulp therapy (VPT), thereby avoiding unnecessary root canal therapy. However, accurately determining the potential for pulp preservation is challenging, and calcification following VPT may complicate future root canal treatment.

Endodontic emergencies due to SIP often involve unscheduled visits, which are inconvenient for patients and disrupt routine clinical schedules. In these situations, the time required for intervention is the primary concern. Incorporating Root ZX3 into the clinical workflow may enable completion of SIP treatment in a significantly shorter timeframe. This efficiency is particularly valuable in emergency settings, as it addresses the immediate pain without necessitating a reduction in chair time for other scheduled patients. Furthermore, by effectively coagulating and incinerating pulp tissue within anatomically complex areas that are often inaccessible to conventional instruments, this technique may mitigate the risks of postoperative pain and related complications. This streamlined procedural approach also serves a dual purpose: it not only minimizes iatrogenic errors, such as ledge formation or file separation caused by the pressure of rushed instrumentation, but also provides the clinician with the necessary time to perform more deliberate access cavity preparation. Such precision is, of course, vital for preserving peri-cervical dentine and preventing perforations of the pulp floor.

## Conclusions

While this study provides valuable preliminary data, its single-arm design and modest sample size of 10 participants naturally restrict the broader applicability of the results. As an exploratory feasibility trial, our focus is on immediate clinical performance rather than establishing long-term definitive evidence. Consequently, the true safety profile and long-term efficacy of Root ZX3 must be validated through future multicenter randomized controlled trials with longer follow-up.

## Appendices

**Table 4:** SPIRIT 2025 checklist

Section / Topic	No	SPIRIT 2025 checklist item description	Reported on page no.
Administrative information			
Title and structured summary	1a	Title stating the trial design, population, and interventions, with identification as a protocol	1
	1b	Structured summary of trial design and methods, including items from the World Health Organization Trial Registration Data Set	2
Protocol version	2	Version date and identifier	3
Roles and responsibilities	3a	Names, affiliations, and roles of protocol contributors	1
	3b	Name and contact information for the trial sponsor	4
	3c	Role of trial sponsor and funders in design, conduct, analysis, and reporting of trial; including any authority over these activities	Not applicable
	3d	Composition, roles, and responsibilities of the coordinating site, steering committee, endpoint adjudication committee, data management team, and other individuals or groups overseeing the trial, if applicable	Not applicable
Open science			
Trial registration	4	Name of trial registry, identifying number (with URL), and date of registration. If not yet registered, name of intended registry	2
Protocol and statistical analysis plan	5	Where the trial protocol and statistical analysis plan can be accessed	6-11
Data sharing	6	Where and how the individual de-identified participant data (including data dictionary), statistical code, and any other materials will be accessible	3-4
Funding and conflicts of interest	7a	Sources of funding and other support (e.g., supply of drugs)	4
	7b	Financial and other conflicts of interest for principal investigators and steering committee members	4
Dissemination policy	8	Plans to communicate trial results to participants, healthcare professionals, the public, and other relevant groups (e.g., reporting in trial registry, plain language summary, publication)	3
Introduction			
Background and rationale	9a	Scientific background and rationale, including summary of relevant studies (published and unpublished) examining benefits and harms for each intervention	4-6
	9b	Explanation for choice of comparator	Not applicable
Objectives	10	Specific objectives related to benefits and harms	6
Methods: Patient and public involvement, trial design			
Patient and public involvement	11	Details of, or plans for, patient or public involvement in the design, conduct, and reporting of the trial	3
Trial design	12	Description of trial design including type of trial (e.g., parallel group, crossover), allocation ratio, and framework (e.g., superiority, equivalence, non-inferiority, exploratory)	5
Methods: Participants, interventions, and outcomes			
Trial setting	13	Settings (e.g., community, hospital) and locations (e.g., countries, sites) where the trial will be conducted	7
Eligibility criteria	14a	Eligibility criteria for participants	7-8
	14b	If applicable, eligibility criteria for sites and for individuals who will deliver the interventions (e.g., surgeons, physiotherapists)	Not applicable
Intervention and comparator	15a	Intervention and comparator with sufficient details to allow replication including how, when, and by whom they will be administered. If relevant, where additional materials describing the intervention and comparator (e.g., intervention manual) can be accessed	7-8
	15b	Criteria for discontinuing or modifying allocated intervention/comparator for a trial participant (e.g., drug dose change in response to harms, participant request, or improving/worsening disease)	9
	15c	Strategies to improve adherence to intervention/comparator protocols, if applicable, and any procedures for monitoring adherence (e.g., drug tablet return, sessions attended)	Not applicable
	15d	Concomitant care that is permitted or prohibited during the trial	9
Outcomes	16	Primary and secondary outcomes, including the specific measurement variable (e.g., systolic blood pressure), analysis metric (e.g., change from baseline, final value, time to event), method of aggregation (e.g., median, proportion), and time point for each outcome	9
Harms	17	How harms are defined and will be assessed (e.g., systematically, non-systematically)	12
Participant timeline	18	Time schedule of enrollment, interventions (including any run-ins and washouts), assessments, and visits for participants. A schematic diagram is highly recommended (see Figure)	6-7
Sample size	19	How sample size was determined, including all assumptions supporting the sample size calculation	7
Recruitment	20	Strategies for achieving adequate participant enrollment to reach target sample size	-
Methods: Assignment of interventions			
Randomization:			
Sequence generation	21a	Who will generate the random allocation sequence and the method used	Not applicable
	21b	Type of randomization (simple or restricted) and details of any factors for stratification. To reduce predictability of a random sequence, other details of any planned restriction (e.g., blocking) should be provided in a separate document that is unavailable to those who enroll participants or assign interventions	Not applicable
Allocation concealment mechanism	22	Mechanism used to implement the random allocation sequence (e.g., central computer/telephone; sequentially numbered, opaque, sealed containers), describing any steps to conceal the sequence until interventions are assigned	Not applicable
Implementation	23	Whether the personnel who will enroll and those who will assign participants to the interventions will have access to the random allocation sequence	Not applicable
Blinding	24a	Who will be blinded after assignment to interventions (e.g., participants, care providers, outcome assessors, data analysts)	Not applicable
	24b	If blinded, how blinding will be achieved and description of the similarity of interventions	Not applicable
	24c	If blinded, circumstances under which unblinding is permissible, and procedure for revealing a participant’s allocated intervention during the trial	Not applicable
Methods: Data collection, management, and analysis			
Data collection methods	25a	Plans for assessment and collection of trial data, including any related processes to promote data quality (e.g., duplicate measurements, training of assessors) and a description of trial instruments (e.g., questionnaires, laboratory tests) along with their reliability and validity, if known. Reference to where data collection forms can be accessed, if not in the protocol	10
	25b	Plans to promote participant retention and complete follow-up, including list of any outcome data to be collected for participants who discontinue or deviate from intervention protocols	11
Data management	26	Plans for data entry, coding, security, and storage, including any related processes to promote data quality (e.g., double data entry; range checks for data values). Reference to where details of data management procedures can be accessed, if not in the protocol	10-11
Statistical methods	27a	Statistical methods used to compare groups for primary and secondary outcomes, including harms	11
	27b	Definition of who will be included in each analysis (e.g., all randomized participants), and in which group	11
	27c	How missing data will be handled in the analysis	11
	27d	Methods for any additional analyses (e.g., subgroup and sensitivity analyses)	Not applicable
Methods: Monitoring			
Data monitoring committee	28a	Composition of data monitoring committee (DMC); summary of its role and reporting structure; statement of whether it is independent from the sponsor and funder; conflicts of interest and reference to where further details about its charter can be found, if not in the protocol. Alternatively, an explanation of why a DMC is not needed	12
	28b	Explanation of any interim analyses and stopping guidelines, including who will have access to these interim results and make the final decision to terminate the trial	12
Trial monitoring	29	Frequency and procedures for monitoring trial conduct. If there is no monitoring, give explanation	12
Ethics			
Research ethics approval	30	Plans for seeking research ethics committee/institutional review board approval	3
Protocol amendments	31	Plans for communicating important protocol modifications to relevant parties	3
Consent or assent	32a	Who will obtain informed consent or assent from potential trial participants or authorized proxies, and how	3
	32b	Additional consent provisions for collection and use of participant data and biological specimens in ancillary studies, if applicable	Not applicable
Confidentiality	33	How personal information about potential and enrolled participants will be collected, shared, and maintained in order to protect confidentiality before, during, and after the trial	10-11
Ancillary and post-trial care	34	Provisions, if any, for ancillary and post-trial care, and for compensation to those who suffer harm from trial participation	-

## References

[1] Levin LG, Law AS, Holland GR, Abbott PV, Roda RS (2009). Identify and define all diagnostic terms for pulpal health and disease states. J Endod.

[2] Berman LH, Hargreaves KM (2021). Cohen's Pathways of the Pulp. ELSEVIER.

[3] (2026). Guide to clinical endodontics. https://www.aae.org/specialty/clinical-resources/guide-clinical-endodontics/.

[4] Gorni FG, Gagliani MM (2004). The outcome of endodontic retreatment: a 2-yr follow-up. J Endod.

[5] Lambrianidis T (2006). Ledging and blockage of root canals during canal preparation: causes, recognition, prevention, management, and outcomes. Endodontic Topics.

[6] Clark D, Khademi J (2010). Modern molar endodontic access and directed dentin conservation. Dent Clin North Am.

[7] Kumagai H, Sugaya T, Tominaga T (2023). Cauterization of narrow root canals untouched by instruments by high-frequency current. Materials (Basel).

[8] Chan AW, Tetzlaff JM, Gøtzsche PC (2013). SPIRIT 2013 explanation and elaboration: guidance for protocols of clinical trials. BMJ.

[9] Glickman GN, Schweitzer JL (2013). Endodontic Diagnosis: Fall 2013 Endodontics: Colleagues for Excellence. https://www.aae.org/specialty/wp-content/uploads/sites/2/2017/07/endodonticdiagnosisfall2013.pdf.

[10] Bergenholtz G, Ricucci D, Siqueira JF (2015). Lesions of endodontic origin. Clinical Periodontology and Implant Dentistry.

[11] Wolters WJ, Duncan HF, Tomson PL (2017). Minimally invasive endodontics: a new diagnostic system for assessing pulpitis and subsequent treatment needs. Int Endod J.

